# A prospective phase II clinical trial of total neoadjuvant therapy for locally advanced gastric cancer and gastroesophageal junction adenocarcinoma

**DOI:** 10.1038/s41598-024-58177-6

**Published:** 2024-03-29

**Authors:** Jin-Ming Shi, Ning Li, Li-Ming Jiang, Lin Yang, Shu-Lian Wang, Yong-Wen Song, Yue-Ping Liu, Hui Fang, Ning-Ning Lu, Shu-Nan Qi, Bo Chen, Ye-Xiong Li, Dong-Bing Zhao, Yuan Tang, Jing Jin

**Affiliations:** 1 Department of Radiation Oncology, National Cancer Center/National Clinical Research Center for Cancer/Cancer Hospital, Chinese Academy of Medical Sciences and Peking Union Medical College, Beijing, 100021 China; 2 State Key Laboratory of Molecular Oncology and Department of Radiology, National Cancer Center/National Clinical Research Center for Cancer/Cancer Hospital, Chinese Academy of Medical Sciences and Peking Union Medical College, Beijing, 100021 China; 3 State Key Laboratory of Molecular Oncology and Department of Medical Oncology, National Cancer Center/National Clinical Research Center for Cancer/Cancer Hospital, Chinese Academy of Medical Sciences and Peking Union Medical College, Beijing, 100021 China; 4 Department of Pancreatic and Gastric Surgery, National Cancer Center/National Clinical Research Center for Cancer/Cancer Hospital, Chinese Academy of Medical Sciences and Peking Union Medical College, Beijing, 100021 China; 5 Department of Radiation Oncology, National Cancer Center/National Clinical Research Center for Cancer/Cancer Hospital & Shenzhen Hospital, Chinese Academy of Medical Sciences and Peking Union Medical College, Shenzhen, 518116 China

**Keywords:** Locally advanced, Gastric cancer, Gastroesophageal junction cancer, Neoadjuvant concurrent chemoradiotherapy, Consolidation chemotherapy, Cancer, Gastroenterology, Oncology

## Abstract

To investigate the safety and efficacy of the neoadjuvant chemoradiotherapy (NCRT) followed by neoadjuvant consolidation chemotherapy (NCCT) and surgery for locally advanced gastric cancer (GC) or gastroesophageal junction (GEJ) adenocarcinoma. Patients diagnosed as locally advanced GC or Siewert II/III GEJ adenocarcinoma with clinical stage T3-4 and/or N positive were prospectively enrolled. Patients underwent NCRT (45 Gy/25 fractions) with concurrent S-1, followed by NCCT (4 to 6 cycles of the SOX regimen) 2 to 4 weeks after NCRT. Gastric cancer radical resection with D2 lymph node dissection was performed 4 to 6 weeks after the total neoadjuvant therapy. The study was conducted from November 2019 to January 2023, enrolling a total of 46 patients. During the NCRT, all patients completed the treatment without dose reduction or delay. During the NCCT, 32 patients (69.6%) completed at least 4 cycles of chemotherapy. Grade 3 or higher adverse events in NCRT (5 cases) were non-hematological. During the course of NCCT, a notable occurrence of hematological toxicities was observed, with grade 3 or higher leukopenia (9.7%) and thrombocytopenia (12.2%) being experienced. A total of 28 patients (60.9%) underwent surgery, achieving R0 resection in all cases. A significant proportion of cases (71.4%) exhibited pathological downstaging to ypT0-2, while 10 patients (35.7%) demonstrated a pathologic complete response (pCR). The total neoadjuvant therapy comprising NCRT followed by NCCT and surgery demonstrates a low severe adverse reactions and promising efficacy, which could be considered as a viable treatment for locally advanced GC or GEJ adenocarcinoma.

**Trial registration:** Clinicaltrials.gov (registration number: NCT04062058); the full date of first trial registration was 20/08/2019.

## Introduction

Gastric cancer (GC) is the fifth frequently diagnosed cancer and the fourth leading cause of cancer-related mortality globally^1^. Surgery remains the cornerstone of curative treatment for non-metastatic gastric cancer. However, even after the curative gastrectomy, a notable 40% to 65% of patients will present locoregional recurrence for locally advanced gastric cancer patients^2^. Although D2 resection can reduce the local and regional recurrence rates in gastric cancer to some extent^3–5^, its worldwide adoption remains limited.

It has been demonstrated that the perioperative chemotherapy could improve survival outcomes; however, the independent utility of chemotherapy strategy in managing locoregional control remains circumscribed^6–8^. The addition of neoadjuvant chemoradiotherapy (NCRT) has been widely adopted to enhance local control and prolong survival in locally advanced gastroesophageal junction (GEJ)^9^. It is reported that patients who achieved pathologic complete response (pCR) have better prognoses^10^. Therefore, we look forward to further enhancing short-term efficacy and long-term survival in patients with locally advanced gastric cancer through neoadjuvant treatment.

In our study, we enrolled gastric cancer and Siewert II/III GEJ adenocarcinoma patients with T3-4 N+ status, as they presented a more challenging prognosis, requiring a more comprehensive and intensive treatment approach. Consequently, in accordance with the intensity-modulated radiotherapy technology and chemotherapy regimens, our primary aim is to assess the safety and effectiveness of a total neoadjuvant approach, encompassing NCRT followed by neoadjuvant consolidated chemotherapy (NCCT) and subsequent surgery for locally advanced gastric cancer and Siewert II/III GEJ adenocarcinoma^11^.

## Methods and materials

### Study design

This prospective clinical phase II trial was designed by the National Cancer Center/Cancer Hospital, Chinese Academy of Medical Sciences (CAMS) and Peking Union Medical College (PUMC) in Beijing, China. It followed the Consolidated Standards of Reporting Trials (CONSORT) reporting guideline^12^. The protocol was approved by ethics committees in our institution and registered at ClinicalTrials.gov (NCT04062058). The written informed consent was obtained from all enrolled patients.

### Eligibility criteria

The main enrolled criteria were listed as followed: (1) aged 18–70 years; (2) karnofsky performance status (KPS) ≥ 70; (3) histologically proven gastric cancer or Siewert II/III GEJ adenocarcinoma; at clinical stage T3–4 and N positive without distant metastases; (4) laboratory requirements were: white blood cell count ≥ 3.0 × 10^9^/L, platelet count ≥ 100 × 10^9^/L, hemoglobin ≥ 10 g/dl, neutrophil count ≥ 1.5 × 10^9^/L; total bilirubin ≤ 1.5 times the upper limit of normal; aspartate aminotransferase and alanine aminotransferase ≤ 1.5 times the upper limit of normal. The exclusion criteria were: age > 70 years or < 18 years, received anticancer treatment before, pregnant or lactation, stage M1 disease. All patients agreed to sign an informed consent form before the enrollment.

### Procedures

#### Neoadjuvant chemoradiotherapy

Patients fasted ≥ 4 h and drank 300 ml semiliquid food 10 min before the four-dimensional computed tomography (4DCT) simulation and radiotherapy to maintain consistent stomach volume. During the simulation, the Computed tomography (CT) scan was conducted, encompassing the clavicle to the fifth lumbar spine, utilizing 0.5 cm slices. Intensity-modulated radiation therapy (IMRT) or volumetric-modulated arc therapy (VMAT) were advised, and subsequently, the radiotherapy dose of 45 Gy was delivered in 25 fractions.

The radiation field encompassed the gross tumor, metastatic lymph nodes, and elective regional lymph nodal stations as determined by the EORTC-ROG and Japan Gastric Cancer Association guidelines^13,14^. The clinical target volume (CTV) comprised the gross tumor volume (GTV), metastatic nodes (GTVnd), and regional lymph nodal stations. According to the ICRU-62 guideline^15^, the planned target volume (PTV) is established by adding 1 cm in the craniocaudal direction and 0.5–0.7 cm in the anterior–posterior and left–right directions to the CTV. Cone beam computed tomography (CBCT) will be conducted daily in the first week then weekly. The concurrent chemotherapy is S-1 will be administered concurrently, with a dosage ranging from 40 to 60 mg taken twice daily, based on the individual body surface area (BSA) (BSA < 1.25 m^2^:40 mg bid; 1.25 m^2^ < BSA < 1.5 m^2^:50 mg bid; BSA > 1.5 m^2^:60 mg bid).

#### Neoadjuvant consolidated chemotherapy

The enhanced CT of the thorax, abdomen, and pelvis will be conducted 2 weeks after the NCRT. For patients without tumor progression, the NCCT process will be performed. The NCCT protocol involves a planned regimen comprising at least 4 to total of 6 cycles of the S-1 and oxaliplatin (SOX) treatment, with each cycle lasting 21 days. On each cycle, patients will be administered intravenous oxaliplatin at a dose of 130 mg/m^2^ on day 1. Concurrently, from day 1 to 14, oral S-1 will be administered at a dose ranging from 40 to 60 mg twice daily according to the BSA. Following the completion of this 14-day treatment phase, there will be a 7-day interval before initiating the subsequent cycle.

In the context of NCRT, if grade 3 gastrointestinal events, neurotoxicity, or grade 4 leukopenia persist for more than 5 days post-treatment without improvement, the treatment dosage will be adjusted to 80% of the initial dose. Additionally, NCCT will be discontinued if there are any grade 4 adverse events, excluding leukopenia.

#### Surgery

Patients will be evaluated by enhanced chest, abdomen and pelvis CT 2 weeks after the last cycle of chemotherapy. If the disease progresses during neoadjuvant therapy, a multiple disciplinary team (MDT) consultation will be required to determine the appropriate course of treatment. For patients suitable for surgery, the R0 radical resection and D2 lymphadenectomy will be recommended. The extent of D2 lymph node dissection is determined based on the location of the tumor^16^.A minimum of 16 lymph nodes must be examined during the surgery. Surgical complications such as anastomotic leakage, anastomotic bleeding or abdominal infection and others within 30 days after surgery will be documented.

### Follow-up

After completing treatment according to the protocol, patients will undergo a comprehensive post-treatment assessment regimen, including physical examinations, blood tests, tumor marker level assessments, imaging scans, and regular follow-up appointments at specified intervals.

### End points

The primary objective of this study is to assess the rate of pCR, which is characterized as the absence of tumor cells in both the primary tumor and the metastatic regional lymph node. The second objectives encompass the evaluation of adverse events during the total neoadjuvant therapy, postoperative complications, the tumor downstaging rate, the rate of achieving a complete resection (R0), the overall survival (OS) and the progression free survival (PFS). Adverse events occurring during treatment were evaluated in accordance with the Common Terminology Criteria for Adverse Events (CTCAE) version 3.0^17^. Tumor regression grade will be assessed utilizing the tumor response grading (TRG) system proposed by Mandard et al.^18^. The OS is characterized as the interval from the enrolled time to mortality resulting from any reasons, and PFS is defined as the interval from the enrolled time to the occurrence of locoregional recurrence, distant metastasis, or death.

### Statistical analyses

Based on the prospective phase II trial conducted in our center, it was observed that the rate of pCR among patients who underwent neoadjuvant chemoradiotherapy was 14%^19^. In this study, it is hypothesized that the pCR rate will increase to 28% following NCRT and NCCT. Considering a 10% dropout rate, the test level α is set at 0.05 with a desired power of 80%. Consequently, the calculated sample size required for this study is 82 patients. Due to a slow patient recruitment rate from November 2019 to September 2023, it has become unfeasible to achieve the targeted sample size. Consequently, the study coordinators have made the decision to terminate patient enrollment by January 2023.

The data are analyzed using SPSS Statistics 22.0 (Chicago, IL, USA). The Kaplan–Meier method and Cox regression model are used to analyze the survival benefit. The Statistical significance will be set to P-value < 0.05.

### Institutional review board statement

The study was conducted in accordance with the Declaration of Helsinki and was approved by Ethics committee of the Chinese Academy of Medical Sciences. (date for the approval: December 10, 2018). The trial is published on ClinicalTrials.gov (number: NCT04062058).

### Informed consent statement

Informed consent was obtained from all subjects involved in the study.

## Results

### Study participants

**Figure 1:**
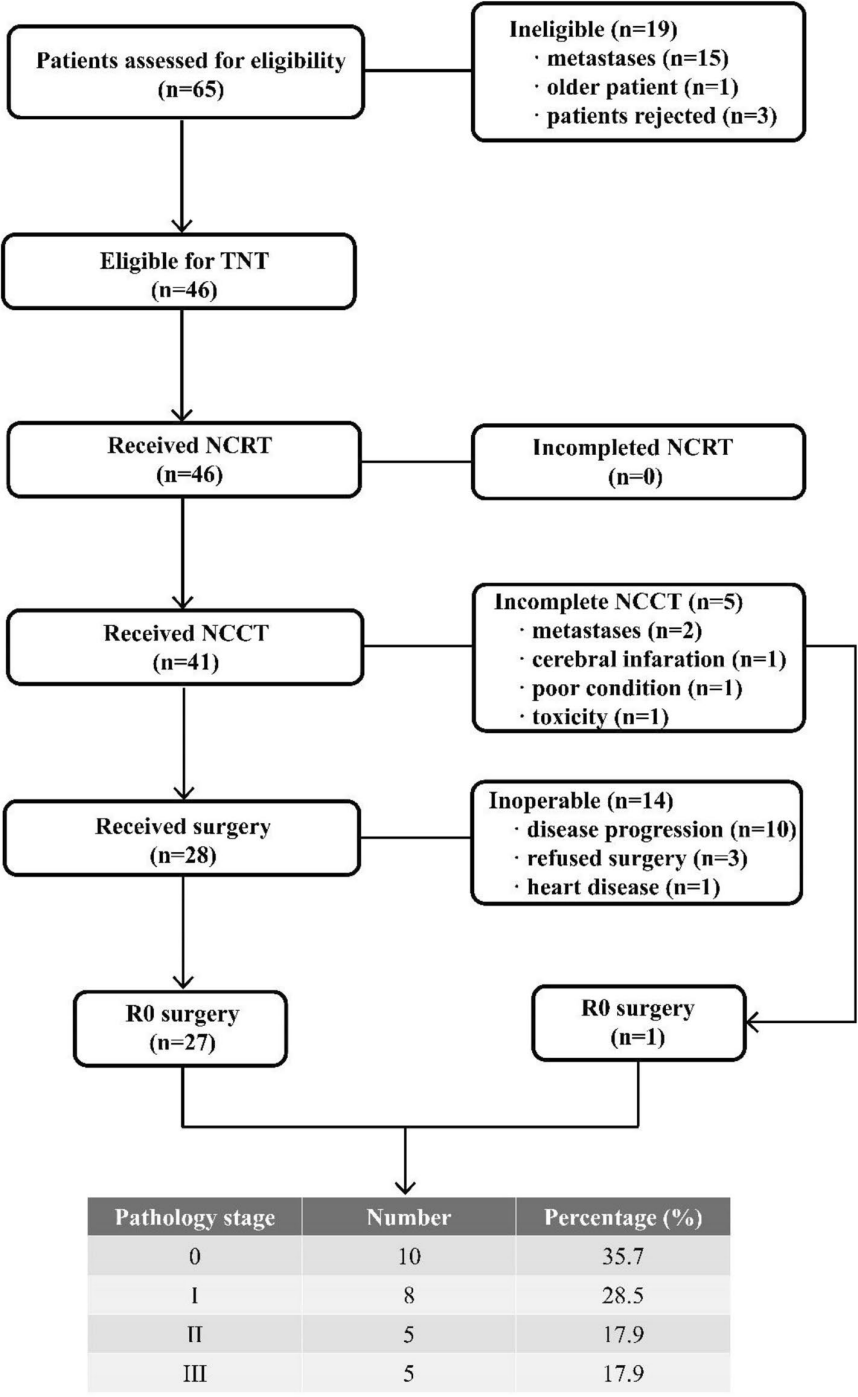
CONSORT diagram. TNT, total neoadjuvant therapy; NCRT, neoadjuvant chemoradiotherapy; NCCT, neoadjuvant consolidated chemotherapy.

A total of 65 patients were screened for the study from November 2019 to January 2023. Among them, 19 patients were excluded based on criteria such as distant metastasis, advanced age, and patient refusal to participate in the clinical research. Eventually, a total of 46 patients were included in the clinical study for evaluation of toxicity, adverse reactions, and treatment compliance. Out of these, 28 patients who underwent surgery were included in the assessment of the primary study endpoints and surgical adverse events. The flowchart of the enrollment process was shown in Fig. 1. The baseline characteristics of the enrolled patients was presented in Table 1. Notably, the majority of the patients were male (80.4%), with a higher incidence of gastroesophageal junction cancer (58.7%), and 87% patients were stage III or stage IVA according to the AJCC 8th edition staging system.

**Table 1:** The baseline characteristics of patients.

Characteristics	N = 46	Percentage (%)	Characteristics	N = 46	Percentage (%)
Age			Lauren type		
Median (range)	53	(22–82)	Intestinal	11	23.9
Gender			Diffuse	7	15.2
Male	37	80.4	Mixed	7	15.2
Female	9	19.6	Undetermined	21	45.7
BMI			HER2 status		
18.5–23.9	24	52.2	Positive	3	6.5
> 24.0	22	47.8	Negative	19	41.3
Signet ring cells			Undetermined	24	52.5
Presence	10	21.7	T-stage		
Absence	36	78.3	T3	7	15.2
Primary site			T4a	35	76.1
Siewert II	11	23.9	T4b	4	8.7
Siewert III	16	34.8	N-stage		
Body	9	19.6	N0	6	13.0
Pylorus	8	17.4	N1	12	26.1
≥ 2/3 of stomach	2	4.3	N2	21	45.7
Differentiation			N3	7	15.2
Well	1	2.2	TNM stage^a^		
Moderate	8	17.4	IIB	6	13.0
Moderate to poorly	10	21.7	III	37	80.4
Poorly	27	58.7	IVA	3	6.5

^a^AJCC 8th.

### Treatment compliance and toxicity

In the intention-to-treat (ITT) population of 46 patients, all patients (100%) received NCRT. After the NCRT, 5 patients (10.9%) didn’t receive NCCT due to distant metastasis (2, 4.3%), poor physical condition (2, 4.3%) or cerebral infarction (1, 2.2%). In the NCCT phase, 9 patients (19.6%) underwent 1–3 cycles of chemotherapy, while the majority of patients (n = 32; 69.6%) adhered to the prescribed regimen and completed at least 4 cycles of chemotherapy.

For the adverse events observed during the total neoadjuvant therapy, during the NCRT, the most common grade 1–2 adverse events were decreased appetite (80.4%), followed by leukopenia (78.3%). Grade 3–4 adverse events were observed in 5 cases, including thrombocytopenia, bleeding, anorexia, nausea, and abdominal pain. During the NCCT, leukopenia was the most frequent adverse events. Additionally, there were 4 cases (9.7%) and 5 cases (12.2%) of patients experiencing grade 3–4 leukopenia and thrombocytopenia, respectively (Table 2). This study reported one case of patient mortality due to hemorrhage following NCRT. This patient underwent multidisciplinary assessment, considering the likelihood of significant bleeding originating from the tumor, staged as T4bN3M0, with deep ulceration observed during gastroscopy before the treatment. During the NCRT, no severe treatment-related adverse events occurred. The major bleeding post-treatment is most likely due to tumor-related factors and cannot be conclusively attributed to NCRT.

**Table 2:** Acute toxicities during the total neoadjuvant therapy.

Toxicities^a^	NCRT N = 46 (%)		CNCT N = 41 (%)	
	Grade 1–2	≥ Grade 3	Grade 1–2	≥ Grade 3
Hematological				
Leukopenia	36 (78.3)	0	30 (73.2)	4 (9.7)
Thrombocytopenia	4 (8.7)	1 (2.2)	9 (21.9)	5 (12.2)
Anemia	7 (15.2)	0	12 (29.3)	0
Non-hematological				
Fatigue	24 (52.1)	0	24 (58.5)	1 (2.4)
Bleeding	0	1 (2.2)	0	0
Diarrhea	1 (2.2)	0	3 (7.3)	0
Anorexia	37 (80.4)	1 (2.2)	26 (63.4)	1 (2.4)
Nausea	32 (69.5)	1 (2.2)	24 (58.5)	3 (7.3)
Vomiting	20 (43.5)	0	10 (24.4)	0
Pain	11 (23.9)	1 (2.2)	4 (9.7)	0
Dermatitis	4 (8.7)	0	0	0
Weight loss	2 (4.3)	0	2 (4.9)	0
Hand-foot syndrome	1 (2.2)	0	4 (9.8)	0

^a^CTCAE version 3.0, NCRT, neoadjuvant chemoradiotherapy; CNCT, concurrent neoadjuvant chemotherapy.

### Pathologic assessment

In the ITT population, a total of 28 patients (60.9%) underwent surgery. The reasons for abstaining from surgery were as followed: 12 cases (26.1%) had tumor progression, 1 case (2.2%) had a heart disease, 2 cases (4.3%) were in poor condition, and 3 cases (6.5%) refused to the surgery. Among patients who received surgery (as shown in Table 3), all 28 patients (100%) successfully underwent R0 resection. One patient (3.6%) underwent D1 resection and the remaining 27 cases (96.4%) underwent D2 resection. Pathologically, 20 patients (71.4%) achieved Mandard scores ranging from 1 to 2, with ypN0 accounting for 67.9%, and ypT0-2 accounting for 71.4%. Pathologic complete response (PCR) was observed in 35.7% of cases. Among the surgical patients, A total of 4 patients (14.3%) experienced postoperative complications, including anastomotic stricture, anastomotic fistula, abdominal infection, and significant bleeding. No surgery-related deaths were observed. In this study, among the 10 patients who did not receive surgery after the NCCT due to tumor progression, 5 patients received palliative chemotherapy, while the other 5 patients chose not to receive any other treatment and opted for observation. For patients received surgery, three patients underwent 2 cycles of postoperative chemotherapy with the SOX regimen. Among these three patients, two had received less than 4 cycles of NCCT, and one patient underwent 4 cycles of chemotherapy before surgery. The remaining patients who underwent surgery did not receive postoperative chemotherapy.

**Table 3:** The surgical outcome for patients received surgery.

	N = 28	Percentage (%)
Number of lymph nodes dissected (median, range)	18	(7–31)
Number of lymph nodes positive (median, range)	0	(0–3)
R0 resection rate	28	100
Mandard score		
TRG1	10	35.7
TRG2	10	35.7
TRG3	5	10.9
TRG4	3	6.5
ypT stage^a^		
T0	10	35.7
T1	4	14.3
T2	6	21.4
T3	7	25.0
T4a	1	3.6
ypN stage^a^		
N0	19	67.9
N1	8	28.6
N2	1	3.6
ypTNM stage^a^		
0	10	35.7
I	8	28.6
II	5	17.9
III	5	17.9

^a^AJCC 8th.

### Survival

The median follow-up time was 25.9 months (range: 1.5 to 62.7 months). In the intention-to-treat (ITT) population of 46 patients, we observed the OS rates at 1, 2, and 3 years were 84.6%, 59.4%, and 47.6%, respectively. The PFS rates for the same group at 1, 2, and 3 years were 67.2%, 53.3%, and 45.2%, respectively. For patients underwent surgery, we found the OS rates at 1, 2, and 3 years were 92.9%, 81.7%, and 62.0%, while, the PFS rates at 1, 2, and 3 years were 89.3%, 70.7%, and 57.6%, respectively. Among the 20 patients who received at least 4 cycles of chemotherapy and surgery, the OS rates at 1, 2, and 3 years were 100%, 90.0%, and 63.0%, respectively, and the PFS rates at 1, 2, and 3 years were 95.0%, 75.0%, and 57.1%, respectively. Through the univariate and multivariate Cox analyses (Table 4), it was determined that patients who underwent surgery significantly improved the PFS and OS compared to those who did not undergo surgery. Additionally, patients who received 4–6 cycles of chemotherapy demonstrated significantly better OS compared to those who received ≤ 3 cycles of chemotherapy.

**Table 4:** Cox analysis for PFS and OS for ITT patients.

Variable	PFS		OS			
	Univariate analysis		Univariate analysis		Multivariate analysis	
	HR (95% CI)	P-value	HR (95% CI)	P-value	HR (95% CI)	P-value
Gender						
Male	1	0.590	1	0.497		
Female	0.779 (0.314–1.933)		0.728 (0.292–1.819)			
Age						
< 60	1	0.847	1	0.725		
≥ 60	1.082 (0.485–2.414)		1.156 (0.514–2.600)			
Location						
GEJ	1	0.178	1	0.157		
Non-GEJ	0.594 (0.278–1.268)		0.570 (0.262–1.241)			
Grade						
Non-poorly differentiated	1	0.058	1	**0.041**	1	0.116
Poorly differentiated	0.058 (0.973–5.050)		2.474 (1.036–5.908)		2.050 (0.838–5.016)	
The cycle of CNCT						
≤ 3 cycles	1	0.058	1	**0.036**	1	**0.026**
4–6 cycles	0.465 (0.211–1.025)		0.426 (0.192–0.945)		0.394 (0.173–0.895)	
Received surgery						
Yes	1	**0.001**	1	**0.004**	1	**0.005**
No	3.787 (1.721–8.333)		3.328 (1.470–7.531)		3.393 (1.453–7.925)	
Clinical TNM stage						
IIB	1		1			
III	0.399 (0.093–1.712)	0.216	0.488 (0.115–2.064)	0.329		
IVA	0.307 (0.088–1.070)	0.064	0.331 (0.095–1.146)	0.081		

*GEJ* gastroesophageal junction, *CNCT* concurrent neoadjuvant chemotherapy.

Significant values are in bold.

## Discussion

In this study, we employed a total neoadjuvant therapy approach involving NCRT, NCCT and surgery. We observed that all patients who underwent surgery achieved R0 resection, with a pCR rate reaching 35.7%. This study provides new evidence for neoadjuvant therapy in the local advanced GC or GEJ cancer.

Under the total neoadjuvant therapy regimen, all patients in this study completed the NCRT, surpassing the completion rate of 66% reported in the POET^20^ study. During the NCCT, 69.9% of patients completed 4–6 cycles, whereas the completion rate was 75% in the POET^20^ study, potentially attributed to the greater number of cycles in our chemotherapy regimen compared to their 3 cycles. The incidence of severe adverse reactions during treatment was relatively low, with non-hematologic toxicities being predominant observed during the NCRT phase. The incidence of severe hematologic reactions, specifically a 12% decrease in white blood cells and a 5% decrease in platelets, were similar to the POET^20^ study. In the TOPGEAR^21^ study's NCRT group, severe gastrointestinal and hematologic adverse reactions occurred at rates of 30% and 52% respectively. Notably, our NCCT regimen involved a greater number of chemotherapy cycles compared to traditional perioperative chemotherapy. During the NCCT neoadjuvant chemotherapy stage in our study, hematologic toxicities were the primary concern, while both the MAGIC^8^ and FLOT4^22^ studies reported adverse reaction rates of over 30%. Overall, the adverse reactions associated with the NCRT combined with NCCT in this study were acceptable.

Regarding the choice of chemotherapy regimen, the concurrent chemoradiotherapy regimen used in this study was S-1. Previous research has shown that S-1 exhibits better tolerability and greater effectiveness than 5-fluorouracil (5-FU) in combined therapy for gastric cancer^23,24^. Furthermore, for the selection of NCCT regimen, this study chose the SOX regimen. The FLOT^22^ regimen is commonly chosen in Europe, while, it exhibited a 27% rate of severe adverse reaction. In the RESOLVE^25^ study conducted in China, it revealed a comparable 3-year progression-free survival rates between postoperative SOX and XELOX regimen. Similarly, the ARTIST II^26^ study demonstrated the superiority of the SOX regimen over S-1. The RESONANCE^27^ study further solidified the SOX regimen as a cornerstone treatment approach. Hence, drawing from the experiences of the aforementioned Phase III studies, this study also adopted the dual-agent SOX regimen for consolidation chemotherapy.

Among the patients who underwent surgery in this study, 100% achieved R0 resection, and 71.4% of patients attained favorable Mandard tumor regression scores. The pCR rate in this study was 35.7%. Stahl et al.^28^ demonstrated a pCR rate of 15.6% in patients with GEJ adenocarcinoma who underwent NCRT. Cats et al.^29^ indicated a pCR rate of 9% in resectable gastric cancer patients who received preoperative chemotherapy. The pCR rate reported in this study surpasses that of current related research. Therefore, the higher pCR rate in this study might be attributed to the treatment approach of radiotherapy followed by chemotherapy, which extended the time from NCRT to surgery. Additionally, consolidation chemotherapy further reduced tumor size.

It is well-known that the association between pCR and survival benefit is currently a matter of debate. The KEYNOTE-585 study^30^ demonstrated that the benefit of pCR does not necessarily translate into improved survival outcomes. However, it is crucial to note that survival benefits often involve multifaceted factors. Moreover, Lorenzen et al.^31^ found that gastroesophageal junction adenocarcinoma patients receiving perioperative chemotherapy and achieving pCR experienced a significantly improved DFS, although no significant difference in OS was observed than those without pCR. Similarly, Li et al.^32^ demonstrated a noteworthy OS benefit for patients achieving pCR with the Sintilimab plus perioperative FLOT regimen, despite the study having a limited sample size. Therefore, whether patients achieving pCR can attain a favorable prognosis requires a comprehensive consideration of various factors. Hence, our study requires continued follow-up to observe patients' prognosis.

Stahl et al.^28^ reported a 3-year OS of 47.4% for gastric cancer patients. In this study, the 3-year OS of the ITT patients and patients who received surgery were 44.2% and 62.0%. It's important to recognize that our study enrolled patients at a later tumor TNM stage and ignore the preoperative abdominal exploration, Additionally, 39.1% of patients were unable to undergo surgery due to tumor progression, which had a discernible impact on the OS rates. However, this also reflects the selective nature of the preoperative treatment for this subset of patients. In the multivariate analysis, receiving 4–6 cycles of chemotherapy and undergoing surgery were favorable prognostic factors for patients. This suggests that completing treatment according to our therapy protocol can effectively improve patient prognosis.

Nevertheless, this study has several limitations: (1) It was a single-arm trial without a randomized controlled experiment against traditional standard treatment and due to the limited follow-up time, we were unable to evaluate the long-term efficacy of the treatment in patients. (2) Preoperative abdominal exploration to confirm the absence of peritoneal metastasis was not conducted, limiting the pretreatment assessment for patients with micro-metastases. However, the utilization of abdominal exploration is not widespread in China^25^. The total neoadjuvant therapy may be beneficial in identifying patients with distant metastases, avoiding the risk of inappropriate treatment due to prior surgery. (3) The completion rate of 4–6 cycles of chemotherapy, particularly 6 cycles, is relatively low. This suggests that 3–4 cycles of preoperative chemotherapy is more appropriate, and the tolerance is more acceptable.

Furthermore, due to the synergy between radiotherapy and immunotherapy^33^, ongoing studies such as SHARED^34^, Neo-PLANET^35^, and PERFECT^36^ are exploring the efficacy of combining immune therapy with NCRT for gastric cancer. We await the results of studies involving these treatment modalities.

## Conclusion

In conclusion, the TNT approach is proven to be feasible, with exceptional prognostic outcomes observed in patients who underwent NCRT, completed 4–6 cycles of NCCT, and surgery. In the future, the application of the TNT model in suitably chosen patient cohorts presents the notable therapeutic effectiveness.

## Data Availability

The datasets used or analyzed during the current study are available from the corresponding author on reasonable request.

## References

[1] Sung H, Ferlay J, Siegel RL (2021). Global cancer statistics 2020: GLOBOCAN estimates of incidence and mortality worldwide for 36 cancers in 185 countries. CA Cancer J. Clin..

[2] D'Angelica M, Gonen M, Brennan MF, Turnbull AD, Bains M, Karpeh MS (2004). Patterns of initial recurrence in completely resected gastric adenocarcinoma. Ann. Surg..

[3] Chang JS, Kim KH, Yoon HI (2017). Locoregional relapse after gastrectomy with D2 lymphadenectomy for gastric cancer. Br. J. Surg..

[4] Sasako M, Sakuramoto S, Katai H (2011). Five-year outcomes of a randomized phase III trial comparing adjuvant chemotherapy with S-1 versus surgery alone in stage II or III gastric cancer. J. Clin. Oncol..

[5] Deng J, Liang H, Wang D, Sun D, Pan Y, Liu Y (2011). Investigation of the recurrence patterns of gastric cancer following a curative resection. Surg. Today..

[6] Choi AH, Kim J, Chao J (2015). Perioperative chemotherapy for resectable gastric cancer: MAGIC and beyond. World J. Gastroenterol..

[7] Ychou M, Boige V, Pignon JP (2011). Perioperative chemotherapy compared with surgery alone for resectable gastroesophageal adenocarcinoma: An FNCLCC and FFCD multicenter phase III trial. J. Clin. Oncol..

[8] Cunningham D, Allum WH, Stenning SP (2006). Perioperative chemotherapy versus surgery alone for resectable gastroesophageal cancer. N. Engl. J. Med..

[9] Shapiro J, van Lanschot JJB, Hulshof MCCM (2015). Neoadjuvant chemoradiotherapy plus surgery versus surgery alone for oesophageal or junctional cancer (CROSS): Long-term results of a randomised controlled trial. Lancet Oncol..

[10] Lin C, Ma J, Zhu C (2023). Is pathologic complete response a good predictor for the long-term, clinical outcome in patients with gastric cancer after neoadjuvant chemotherapy? A retrospective, multi-institution study in China. Ann. Surg. Oncol..

[11] Siewert JR, Stein HJ (1998). Classification of adenocarcinoma of the oesophagogastric junction. Br. J. Surg..

[12] Moher D, Hopewell S, Schulz KF (2010). CONSORT 2010 explanation and elaboration: Updated guidelines for reporting parallel group randomised trials. BMJ.

[13] Matzinger O, Gerber E, Bernstein Z (2009). EORTC-ROG expert opinion: Radiotherapy volume and treatment guidelines for neoadjuvant radiation of adenocarcinomas of the gastroesophageal junction and the stomach. Radiother. Oncol..

[14] Associations JGC (2011). Japanese classification of gastric carcinoma: 3rd English edition. Gastric Cancer..

[15] Stroom JC, Heijmen BJ (2002). Geometrical uncertainties, radiotherapy planning margins, and the ICRU-62 report. Radiother. Oncol..

[16] [Chinese expert consensus on extent of standardized lymphadenectomy for locally advanced gastric cancer (2022 edition)]. *Zhonghua Wei Chang Wai Ke Za Zhi*. **25**(4), 277–283 (2022). 10.3760/cma.j.cn441530-20220322-00111. 10.3760/cma.j.cn441530-20220322-00111 35461192

[17] Cancer Therapy Evaluation Program: Common Terminology Criteria for Adverse Events (CTCAE). https://ctepcancergov/protocolDevelopment/electronic_applications/ctchtm. 10.7150/jca.41950.

[18] Mandard AM, Dalibard F, Mandard JC (1994). Pathologic assessment of tumor regression after preoperative chemoradiotherapy of esophageal carcinoma. Clinicopathologic correlations. Cancer..

[19] Wang X, Zhao DB, Yang L (2022). Preoperative concurrent chemoradiotherapy versus neoadjuvant chemotherapy for locally advanced gastric cancer: Phase II randomized study. Front. Oncol..

[20] Stahl M, Walz MK, Riera-Knorrenschild J (2017). Preoperative chemotherapy versus chemoradiotherapy in locally advanced adenocarcinomas of the oesophagogastric junction (POET): Long-term results of a controlled randomised trial. Eur. J. Cancer..

[21] Leong T, Smithers BM, Haustermans K (2017). TOPGEAR: A randomized, Phase III trial of perioperative ECF chemotherapy with or without preoperative chemoradiation for resectable gastric cancer: Interim results from an international, intergroup trial of the AGITG, TROG, EORTC and CCTG. Ann. Surg. Oncol..

[22] Al-Batran S-E, Homann N, Pauligk C (2019). Perioperative chemotherapy with fluorouracil plus leucovorin, oxaliplatin, and docetaxel versus fluorouracil or capecitabine plus cisplatin and epirubicin for locally advanced, resectable gastric or gastro-oesophageal junction adenocarcinoma (FLOT4): A randomised, phase 2/3 trial. Lancet..

[23] Ajani JA, Winter K, Komaki R (2008). Phase II randomized trial of two nonoperative regimens of induction chemotherapy followed by chemoradiation in patients with localized carcinoma of the esophagus: RTOG 0113. J. Clin. Oncol..

[24] Boku N, Yamamoto S, Fukuda H (2009). Fluorouracil versus combination of irinotecan plus cisplatin versus S-1 in metastatic gastric cancer: A randomised phase 3 study. Lancet Oncol..

[25] Zhang X, Liang H, Li Z (2021). Perioperative or postoperative adjuvant oxaliplatin with S-1 versus adjuvant oxaliplatin with capecitabine in patients with locally advanced gastric or gastro-oesophageal junction adenocarcinoma undergoing D2 gastrectomy (RESOLVE): An open-label, superiority and non-inferiority, phase 3 randomised controlled trial. Lancet Oncol..

[26] Park SH, Lim DH, Sohn TS (2021). A randomized phase III trial comparing adjuvant single-agent S1, S-1 with oxaliplatin, and postoperative chemoradiation with S-1 and oxaliplatin in patients with node-positive gastric cancer after D2 resection: The ARTIST 2 trial. Ann. Oncol..

[27] Wang X, Li S, Xie T, Lu Y, Guo X, Lin C (2020). Early results of the randomized, multicenter, controlled evaluation of S-1 and oxaliplatin as neoadjuvant chemotherapy for Chinese advanced gastric cancer patients (RESONANCE Trial). J. Clin. Oncol..

[28] Stahl M, Walz MK, Stuschke M (2009). Phase III comparison of preoperative chemotherapy compared with chemoradiotherapy in patients with locally advanced adenocarcinoma of the esophagogastric junction. J. Clin. Oncol..

[29] Cats A, Jansen EPM, van Grieken NCT (2018). Chemotherapy versus chemoradiotherapy after surgery and preoperative chemotherapy for resectable gastric cancer (CRITICS): An international, open-label, randomised phase 3 trial. Lancet Oncol..

[30] Shitara K, Rha SY, Wyrwicz LS (2024). Neoadjuvant and adjuvant pembrolizumab plus chemotherapy in locally advanced gastric or gastro-oesophageal cancer (KEYNOTE-585): An interim analysis of the multicentre, double-blind, randomised phase 3 study. Lancet Oncol..

[31] Lorenzen S, Thuss-Patience P, Al-Batran SE (2013). Impact of pathologic complete response on disease-free survival in patients with esophagogastric adenocarcinoma receiving preoperative docetaxel-based chemotherapy. Ann. Oncol..

[32] Li N, Li Z, Fu Q (2024). Efficacy and safety of neoadjuvant sintilimab in combination with FLOT chemotherapy in patients with HER2-negative locally advanced gastric or gastroesophageal junction adenocarcinoma: An investigator-initiated, single-arm, open-label, phase II study. Int. J. Surg..

[33] Zhang Z, Liu X, Chen D, Yu J (2022). Radiotherapy combined with immunotherapy: The dawn of cancer treatment. Signal Transduct. Target Ther..

[34] Wei J, Lu X, Liu Q (2022). Efficacy and safety of sintilimab in combination with concurrent chemoradiotherapy for locally advanced gastric or gastroesophageal junction (GEJ) adenocarcinoma (SHARED): Study protocol of a prospective, multi-center, single-arm phase 2 trial. Cancer Manag. Res..

[35] Tang Z, Wang Y, Liu D (2022). The Neo-PLANET phase II trial of neoadjuvant camrelizumab plus concurrent chemoradiotherapy in locally advanced adenocarcinoma of stomach or gastroesophageal junction. Nat. Commun..

[36] van den Ende T, de Clercq NC, van Berge Henegouwen MI (2021). Neoadjuvant chemoradiotherapy combined with atezolizumab for resectable esophageal adenocarcinoma: A single-arm phase II feasibility trial (PERFECT). Clin. Cancer Res..

